# Simulation TRaining for Operative vaginal Birth Evaluation: study protocol for an observational stepped-wedge interrupted time-series study (STROBE)

**DOI:** 10.1186/s12884-019-2222-x

**Published:** 2019-04-02

**Authors:** Stephen O’Brien, Erik Lenguerrand, Sharon Jordan, Katie Cornthwaite, Christy Burden, Laura Timlin, Dimitrios Siassakos

**Affiliations:** 1 Translational Health Sciences, University of Bristol, Canynge Hall, 39 Whatley Road, Bristol, BS8 2PS UK; 2 Women & Children’s Directorate, North Bristol NHS Trust, Bristol, UK; 3 Research & Innovation, North Bristol NHS Trust, Bristol, UK

**Keywords:** Training, Birth, Operative vaginal birth, Forceps, Ventouse, Outcomes

## Abstract

**Background:**

Operative vaginal birth is a common procedure used to expedite birth after full cervical dilatation where there is a clinical need to do so (15% of births in the UK in 2016). The acquisition of skills for operative vaginal birth is dependent on the exposure of junior obstetricians to situations in which they can undertake directly supervised learning from senior accouchers. The Royal College of Obstetricians and Gynaecologists has recently introduced the first structured course in operative vaginal birth. To date, there have been no attempts to determine the clinical impact of a structured training package for operative vaginal birth.

**Methods:**

The STROBE study is a quasi-experimental before-after interrupted time-series study of the effect of simulation training in operative vaginal birth for obstetricians on clinical outcomes of women and babies following operative vaginal birth. Similar to a stepped-wedge design, the intervention will be gradually implemented in all participating units but at different time periods. The primary outcome is failed operative vaginal birth with the first intended instrument. Secondary maternal outcomes are; use of second instrument to achieve operative vaginal birth, caesarean section, episiotomy, perineal trauma (1st, 2nd, 3rd, 4th degree tear), cervical tear requiring suturing, general anaesthesia and estimated blood loss. Secondary neonatal outcomes are; Apgar score at one, five, and ten minutes, Umbilical artery pH, shoulder dystocia, admission to Neonatal Intensive Care Unit and death within 28 days of birth. The analysis will be intention-to-treat (per unit) on the primary and secondary outcomes. The STROBE study received approval from the Health Research Authority and is sponsored by North Bristol NHS Trust. Results will be published in an open-access peer-reviewed medical journal within one year of completion of data gathering.

**Discussion:**

The STROBE study will help establish our understanding of the effectiveness of locally-delivered simulation training for operative vaginal birth. Robust evidence supporting the effectiveness of such an approach would add weight to the argument supporting regular, local training for junior obstetricians in operative vaginal birth.

**Trial registration:**

ISRCTN11760611 05/03/2018 (retrospectively registered).

**Electronic supplementary material:**

The online version of this article (10.1186/s12884-019-2222-x) contains supplementary material, which is available to authorized users.

## Background

Complications of the second stage of labour (fetal distress, obstructed labour, maternal exhaustion or medical condition requiring shortening of the second stage of labour) remain a major cause of maternal and neonatal mortality and morbidity across the world – such complications are responsible for 4 to 13% of maternal deaths in Africa, Asia, Latin America and the Caribbean [1], and in 2013 obstructed labour alone accounted for 0.4 deaths per 100,000 women worldwide [2].

These complications can be mitigated by the accoucheur performing either an operative vaginal birth (OVB) or a caesarean section - when performed in an appropriate setting by skilled accoucheurs, OVB can reduce adverse outcomes for women and their babies relative to caesarean section [3], and remains a useful and viable strategy for the management of complications in the second stage of labour [4].

OVB is a complex skill that requires an understanding of the anatomy, constant re-evaluation of the situation, fine motor skills that respond to haptic feedback, and continuous simultaneous communication with both professional colleagues and the patient – axiomatically, this takes time to learn [5]. At present, the majority of useful learning by junior accoucheurs is conducted via ‘learning-on-the-job’, performing either parts or whole OVBs under direct, often hands-on supervision from a senior accoucheur [6].

Various attempts have been made in different settings to standardise and improve maternal and neonatal outcomes in OVB, including the provision of dedicated senior labourists [7, 8] and training junior accouchers in the use of forceps prior to the use of ventouse [9]. While these have shown increases in the rate of OVB performed, no changes in maternal or neonatal outcomes have yet been demonstrated. Moreover, no studies have examined the impact of attempts to increase practitioner skills and thereby clinical outcomes in more than one setting – all previous studies have been limited to locally implemented strategies alone.

In response to this clear need to provide structured OVB skills training to junior accouchers, the Royal College of Obstetricians and Gynaecologists (RCOG) has developed a structured training course to ensure provision of OVB skills to junior accouchers during their training, the RCOG Operative Birth Simulation Training course, (ROBuST). In 2017 the ROBuST course was introduced as a compulsory part of the speciality training curriculum for junior obstetricians in the UK. However, the impact of this course has not been evaluated. Our study seeks to determine the impact of structured training in OVB for junior accouchers on routinely collected maternal and neonatal outcomes across 4 large maternity units (Structured Training in Operative vaginal BirthE – the STROBE study).

## Methods

### Study design

This is a quasi-experimental before-after interrupted time-series study investigating routinely collected maternal and neonatal outcomes after OVB covering a time period which includes delivery of local simulation courses in OVB. Details of the study design are presented in Table 1. Similar to a stepped-wedge design, the intervention, local simulation courses in OVB, will be gradually implemented in all participating units with outcome measures collected before and after the implementation of the intervention. No data is collected for the time-period during which the training will be implemented. Randomisation of the training implementation period is not possible due to the participating units requiring the training intervention at specific times.

**Table 1:**
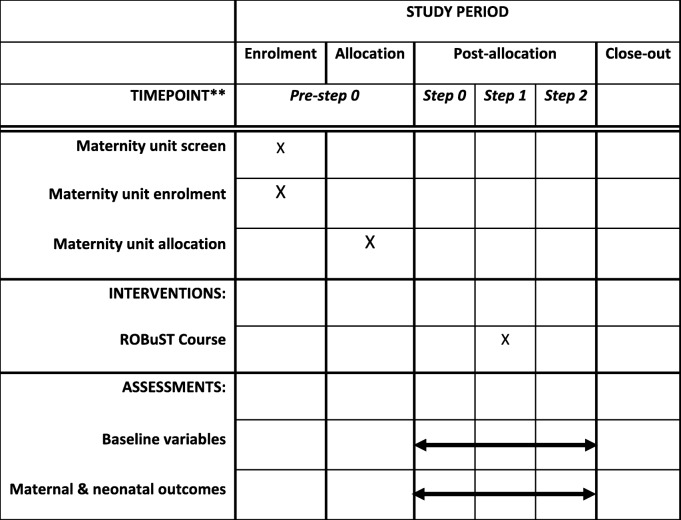
SPIRIT diagram for the STROBE Study

If a participating unit is unable to implement the intervention, it will be considered as a control site allowing us to account for any potential change, independent of our study, in the clinical context surrounding OVB and its management. The STROBE guidelines were adhered to in the design of this study.

### Intervention studied

The intervention studied will be the local provision of structured simulation training in operative vaginal birth (the ROBuST course) to trainees in O&G in the South West of England. The intervention will be delivered by local faculty of senior obstetricians and midwives. The ROBuST course is a one-day course that utilises simulation models to teach the spectrum of operative birth manoeuvres – rotational and non-rotational forceps and vacuum deliveries, as well as techniques for complex caesarean sections.

### Participants

The study population will consist of women and babies having an attempted OVB in the four maternity units during the study period (12 months per site, between 700 to 850 women per site, at least 3000 women in total).

### Inclusion criteria

Data will be included in data collection and analysis if all of the following apply:

- A vaginal birth was conducted within a study site during the applicable study time period
- An operative vaginal birth instrument (forceps or vacuum) was applied to a fetal head

### Exclusion criteria

Data from births will not be included in collection and analysis if any of the following apply:

- An operative birth instrument was only applied during (and not before) a caesarean section (i.e. use of Wrigley’s forceps at caesarean)
- The woman had a multiple pregnancy (twins or higher)
- If the woman is < 18 years old at the time of birth
- If the woman is a prisoner

### Time period of outcomes studied

12 months. Of this, data will be collected for three to 6 months prior to each unit of trainees attending OVB simulation training and four to 6 months after. This equates to 12 months of outcomes studied per site, due to variable data collection lengths of different sites. The time period of the training intervention will be two to 3 months, depending on the ability of participating units to implement training at higher frequencies to train all of their individual doctors.

Data will not be collected during the training intervention period. Doctors within units will be trained at different rates within the training periods, and so no comparison can be made between training periods between units as at any given time different proportions of doctors will have been exposed to training.

### Primary outcome measure

The primary outcome measure will be failed operative vaginal birth with first chosen instrument. This has been chosen as failure with the first instrument will lead to either use of a second instrument or reversion to caesareans section – both of which have been demonstrated to be independently associated with poorer outcomes than success with the first instrument [3, 10]. Moreover, this is the chosen primary outcome of the most recent Cochrane review of the subject [11].

### Secondary outcome measures

The following secondary maternal outcomes measures will be collected; use of second instrument to achieve OVB, caesarean section, episiotomy, perineal trauma (1st, 2nd, 3rd, 4th degree tear), cervical tear requiring suturing, general anaesthesia and estimated blood loss (EBL). The following secondary neonatal outcomes will be collected; Apgar score at 1, 5, 10 min, Umbilical artery pH, shoulder dystocia, admission to Neonatal Intensive Care Unit (NICU), death within 28 days of birth.

### Clinical variable characteristics

The following clinical variable characteristics will be collected and used for adjusting and comparing secondary clinical outcomes; maternal age (years), body mass index (BMI) at booking, parity, history of previous caesarean or vaginal birth, length of gestation (completed weeks), duration of first and second stage (minutes), indication for assisted vaginal birth (presumed fetal compromise, delay in 2nd stage, maternal exhaustion, other), rotation required as part of OVB (Yes/No), analgesia (epidural block, spinal block, general anaesthesia, pudendal block, perineal infiltration, none), baby birth weight (g), grade of operator (Speciality Trainee (years of residency in O&G) (ST) 1–2, ST 3–5, ST 6–7, Consultant), grade of supervisor (if applicable, ST 3–5, ST 6–7, Consultant).

### Process measures

The following will be recorded as process measures; number and proportion (%) of trainees exposed to intervention per site during training period, number and seniority of local facilitators of intervention.

### Frequency of primary outcome measure and expected measure of effect

The primary outcome is failed OVB. Depending on the type of OVB, rates of failure in reported studies vary between 5.8% (rotational forceps) [12, 13], 9.3% (non-rotational forceps), 14.1% (all types of ventouse) and 24.4% (hand-held ventouse) [11].

We propose to take 80% as an estimated real-world success rate (this is lower than that reported in other studies, as OVBs reported in studies will be subject to the Hawthorne effect. We also seek to reflect the mix of types of OVB performed).

### Sample size

We have generated the following study power calculations scenarios using sample size formulae for stepped-wedge design proposed by Hussey et al. [14] and implemented in Stata by Hemming et al. [12]. We have used a two-sided alpha of 0.05 and assumed an inter-cluster co-efficient (ICC) of 0.1.

### Conservative estimate

Assuming 4 units, each exposed to the intervention at a different step (Table 2), i.e. one unit per step, each step of 3 months length, at least one observation period prior to the implementation of the intervention and one period after its implementation, and no data collected during the intervention implementation (washout-period) and 200 births per unit per period/step, the study will be able to detect a change from 20 to 10% in the proportion of failed OVB with a 76% power.

**Table 2:** Stepped-wedge interrupted time series design of STROBE study

	Step 0	Step 1	Step 2	Step 3	Step 4	Step 5
Unit 1	Pre-training data collection period	Intervention implemented, no data collected	Post-training data collection period	Post-training data collection period	Post-training data collection period	Post-training data collection period
Unit 2	Pre-training data collection period	Pre-training data collection period	Intervention implemented, no data collected	Post-training data collection period	Post-training data collection period	Post-training data collection period
Unit 3	Pre-training data collection period	Pre-training data collection period	Pre-training data collection period	Intervention implemented, no data collected	Post-training data collection period	Post-training data collection period
Unit 4	Pre-training data collection period	Pre-training data collection period	Pre-training data collection period	Pre-training data collection period	Intervention implemented, no data collected	Post-training data collection period

### Optimistic estimate

Using the same set of hypotheses but assuming 266 births per unit per period, the same change would be detected with a power of 89%.

Based on the delivery figures for the 4 units for 2016–2017, around 250 (eligible) deliveries are expected per three-month periods, suggesting that for a 12 month period (defined as per funding constraint and to limit contamination due to trainees rotating between hospitals), we expect the power to be between 76 and 89% to detect a 50% reduction in failed OVB from 20 to 10%.

### Data collection

Study data will take the form of electronic and paper-based clinical patient notes at each study site. Data will be extracted and uploaded following collation onto a study-specific iteration of a secure electronic database hosted by the University of Bristol (*REDcap*) using password-protected NHS computers.

All data held on secure computing networks (both NHS and University of Bristol) will be protected by using a combination of passwords and file permissions.

All files, paper and electronic data will be transferred to secure archiving no more than 3 years after the end of the study. Data will be stored for 5 years after the study is complete, in line with the MRC Guidance on Personal Information in Medical Research [13]. Data procedures will be in keeping with the stipulations in the Data Protection Act 2000.

### Data analysis

#### Participation, loss to follow-up and withdrawal

While the current study design is not a formal stepped-wedge study design, and has no randomisation component, its analysis and reporting will respect the principles of the CONSORT guideline [15] and available guidelines for stepped-wedge design at the time of analysis. Unit recruitment, in-house trainers’ participation in training and in-house training implementation will be documented as frequency and proportions.

Loss to follow-up will only occur if a maternity unit is closed. To our knowledge, no unit closures or merging are planned for the duration of the project.

#### Baseline and intervention description

The total and unit-specific number of births (count), OVB (count), failed attempted OVB (count) and rate of failed attempted OVB (%) will be tabulated by step/period (zero to five).

The frequency and proportion of staff trained and number of training sessions delivered by in-house trainers will also be reported for each of the 4 units. Proportions, means with standard deviation or median with inter-quartile range will be reported as appropriate to describe the maternal and neonatal outcomes.

#### Main analysis

The outcome for the main analysis is the rate of successful attempted OVB in term infants, dichotomised into successful or failed. This dichotomous outcome measured for each birth will be analysed with a modified Poisson regression (with robust estimation of the variance) [16]. All models will be adjusted for hospital units as fixed to account for clustering at maternity unit level, i.e. correlation between births occurring in the same maternity unit. A formal mixed or marginal regression model cannot be considered due to the reduced number of hospital units involved in this study (n = four).

In the main analysis, we will assess the intervention effect (control periods vs. post-intervention periods) and include an adjustment for the time period (period zero to five) to account for any underlying time trend susceptible to confound the assessment of the main effect [17, 18]. We will either model the time periods with categorical fixed effect variables (with dummy indicators for each period) or as a continuous factor with appropriate polynomial function of time if required. Statistics such as the Aikeke information criterion will be used to select the best modelling for time.

The interaction between time and the main intervention effect will then be modelled to investigate the timing and duration of the intervention effect. The study is not powered for such interaction and this last model should be considered as exploratory.

We will use the Log-likelihood ratio or Wald test and *p*-value of 0.05 to assess the strength of the association, assuming that any p-value equal or below 0.05 (two-sided) will show evidence of an association between the intervention and outcome.

No interim analysis is planned.

The model will then be adjusted for patient, delivery or hospital characteristics susceptible to influence the primary or secondary outcome such maternal BMI, length of second stage of labour and fetal weight. These analyses will be conducted on an intention-to-treat principle, i.e. a unit will be considered as exposed to the intervention 3 months after the initial planed date of intervention implementation.

#### Sensitivity analyses

To account for any departure from the planned and scheduled intervention, i.e. for units which do not (fully) comply with or do not implement the intervention, a sensitivity analysis will test the effect of the intervention using the actual date of the intervention was first implemented. These analyses will be called “as implemented”. For units not compliant with the implementation schedule, births performed prior to the actual start of the intervention will be considered as “not exposed”, while births performed 3 months after the start of the first training session will be considered as performed during the “exposed” period. The same modelling strategy described in the previous section will be used.

We will also investigate the possibility to extend the analyses to the time-periods prior to this project to include a longer control period.

We do not anticipate that there will be extensive missing data for the primary outcome and our primary analysis strategy will be on complete cases. However, it is known that for a small number of births the attempt to perform an OVB is not collected. The main analyses will be conducted on complete-cases. We will then describe any missing data in detail, and if required, will test the robustness of our primary analysis using three approaches; all births with missing data will be considered as failed OVB, they will then be considered as successful OVB, and finally multiple imputations by chained equation will be conducted.

#### Additional analyses

The same modelling strategy will be used for all secondary maternal and perinatal outcomes. Appropriate generalised linear model (linear, modified Poisson, multinomial logistic regression model) will be used depending on the distributions of the considered outcome. Continuous outcomes will be modelled with linear regression on their raw or transformed scale if the residuals of the model are not normality distributed. They might need to be categorised if no appropriate transformation is found and it will be done in respect with the literature. The Apgar scores at 5 minutes will be dichotomised into < 7 or ≥ 7, postpartum haemorrhage (PPH) dichotomised into < 1000 ml or ≥ 1000 ml. and umbilical arterial pH dichotomised into < 7.2 and ≥ 7.2.

### Safety reporting

This is an observational project, and as such no reports will be produced until after full data has been gathered and analysed. This project involves the reviewing of a substantial number of clinical records in order to extract outcome data. It is therefore likely that untoward clinical incidents will be encountered. Should a potential clinical incident be encountered, the study team will inform the Patient Safety Midwife at the relevant study site, via secure NHSmail. This message will include the full patient details and a brief description of the incident. The Patient Safety team will then undertake an initial review of the incident and will escalate to a formal Patient Safety Investigation if this is required. This would involve informing the patient that such an investigation is underway.

It is likely that the majority of these potential incidents will already have been investigated by the Patient Safety team.

The potential clinical incidents which will be notified to the Patient Safety teams are; PPH ≥ 3000 ml, 4th degree anal sphincter tear.

### Ethics and dissemination

Women are at the heart of this study – this study will be conducted ‘with women’, rather than simply ‘on women’. Patient and public involvement (PPI) has been incorporated into this study at several keys stages – ethical justification and dissemination.

This study involves the retrospective reviewing of patient case notes for anonymised data extraction without consent. While this is justifiable due to the nature of outcome data required and the study resources, the study team recognises that this course of action needs to be justifiable with women specifically. Therefore, the study team took part in a round of PPI to specifically address this issue – Additional file 1. This round of PPI confirmed that women are amenable to this approach, provided that strong safeguards regarding management of uncovered clinical incidents are in place. In order to facilitate woman-facing dissemination of the study results, this study will develop a Communication Plan to disseminate the results in consultation with local women’s groups. A dissemination plan has been drawn up by the SSG (including input from a patient representative) and following commencement of the study, will then be circulated to a North Bristol NHS Trust convened panel of maternity service users for input. Following this input, the plan will be actioned, with the intention that much of the public-facing roles will be taken by interested women themselves.

The study is sponsored by North Bristol NHS Trust. Data collection is ongoing as of July 2018. Locality approvals for the collection of data have been secured prior to the end of study point of 31st August 2018. Changes to the protocol will be sought as required, incorporated into trial registration and communicated to study sites once secured.

We will publish the results of the study within a peer-reviewed medical journal within 1 year of completion of data gathering. The results will be disseminated to the study sites, the sponsor and the RCOG.

## Discussion

OVB is one of the most common obstetrical interventions, and yet there are no interventions which have been demonstrated to improve outcomes for women and babies on a more than local level. This study seeks to evaluate a generalisable training program that is being implemented nationwide within the UK. Should the results prove positive, it will provide robust evidence for the provision of such training. If the results are equivocal or negative, it should prompt re-evaluation and re-design of any such training program in the future.

## Additional file

Additional file 1: STROBE PPI. Responses of patients and public to suggested design of STROBE study. (DOCX 82 kb)

## Abbreviations

BMI: Body Mass Index
EBL: Estimated Blood Loss
HRA: Health Research Authority
ICC: inter-cluster co-efficient
NICU: Neonatal Intensive Care Unit
OVB: Operative Vaginal Birth
PPH: Postpartum Haemorrhage
PPI: Patient and Public Involvement
RCOG: Royal College of Obstetricians and Gynaecologists
SSG: Study Steering Group
ST: Speciality Trainee

## References

[1] Khan KS, Wojdyla D, Say L, Gülmezoglu AM, Van Look PF (2006). WHO analysis of causes of maternal death: a systematic review. Lancet.

[2] GBD 2013 Mortality and Causes of Death Collaborators (2015). Global, regional, and national age-sex specific all-cause and cause-specific mortality for 240 causes of death, 1990–2013: a systematic analysis for the Global Burden of Disease Study 2013. Lancet.

[3] Murphy DJ, Liebling RE, Verity L, Swingler R, Patel R (2001). Early maternal and neonatal morbidity associated with operative delivery in second stage of labour: a cohort study. Lancet.

[4] Draycott TJ, Di Renzo GC (2017). The role of operative vaginal birth in the 21st century and a way forward. BJOG.

[5] Attilakos G, Draycott T, Gale A, Siassakos D, Winter C. ROBuST: RCOG operative birth simulation training. London: Cambridge University Press; 2013.

[6] Gale A, Siassakos D, Attilakos G, Winter C, Draycott T (2014). Operative vaginal birth: better training for better outcomes. BJOG.

[7] Solt I, Jackson S, Moore T, Rotmensch S, Kim MJ (2011). Teaching forceps: the impact of proactive faculty. Am J Obstet Gynecol Elsevier Inc.

[8] Hardy C, Roberts R (2014). The role of rotational forceps. BJOG.

[9] Skinner S, Davies-Tuck M, Wallace E, Hodges R (2017). Perinatal and maternal outcomes after training residents in forceps before vacuum instrumental birth. Obstet Gynecol.

[10] Murphy DJ, Macleod M, Bahl R, Strachan B (2011). A cohort study of maternal and neonatal morbidity in relation to use of sequential instruments at operative vaginal delivery. EJOG Elsevier Ireland Ltd.

[11] O’Mahony F, Hofmeyer GJ, Menon V, O’Mahony F (2010). Choice of instruments for assisted vaginal delivery.

[12] Hemming K, Girling A (2014). A menu-driven facility for power and detectable-difference calculations in stepped-wedge cluster-randomized trials. Stata J.

[13] Medical Research Council Guidance. Personal information in medical research. 2nd ed. London: Medical Research Council; 2000. p. 1–48.

[14] Hussey MA, Hughes JP (2007). Design and analysis of stepped wedge cluster randomized trials. Contemp Clin Trials.

[15] Schulz KF, Altman DG, Moher D, for the CONSORT group. CONSORT 2010 statement: updated guidelines for reporting parallel group randomised trials. BMJ. 2010;340(mar23 1):c332–2. 10.1136/bmj.c332 PMC2844940 20332509

[16] Zou G (2004). A modified poisson regression approach to prospective studies with binary data. Am J Epidemiol.

[17] Hemming K, Taljaard M, Forbes A (2017). Analysis of cluster randomised stepped wedge trials with repeated cross-sectional samples. Trials 3rd ed.

[18] Barker D, D’Este C, Campbell MJ, McElduff P (2017). Minimum number of clusters and comparison of analysis methods for cross sectional stepped wedge cluster randomised trials with binary outcomes: a simulation study. Trials.

