# Supplementary material for: Simulation TRaining for Operative vaginal Birth Evaluation: study protocol for an observational stepped-wedge interrupted time-series study (STROBE)
Source: BMC Pregnancy Childbirth. 2019 Apr 2;19:109. doi: 10.1186/s12884-019-2222-x (PMC6444670; doi:10.1186/s12884-019-2222-x)
Supplement: Supplementary file 1 — STROBE PPI. Responses of patients and public to suggested design of STROBE study. (DOCX 82 kb) [file 12884_2019_2222_MOESM1_ESM.docx]

**Patient and Public Involvement in Research**

**Summary of patient responses to the STROBE study**

**Responses collected ante-natal women following attendance at ante-natal assessment unit on 23^rd^ August 2017**

**All participants are women who are currently pregnant and anticipating a vaginal birth**

**Prior to discussion the women were presented with a brief precis of the STROBE study**

**Discussion question:** What would your views be if we (the STROBE research team) carried out a review of patient’s notes following birth, to gain knowledge about outcomes and the process of our systems and training, along with the type of birth you had, and how the care was delivered?  Do you think it would be fair and feel that it is ethical to review such a batch of notes without contacting all of the individuals first?

**Participant 1**:  ST

“That would be fine, I have no real feelings one way or the other.  I think it is good that someone is bothering to look into these things.”

**Participant 2**:  RG

“I think that is fine, as long as that person’s details are kept confidential, and not banded around.  But If any harm issues were found, I would hope that would be communicated to the individual.”

**Participant 3**:  LB

“I would just be glad people care and everything is being looked into!”

**Summary**

Participants (representatives of the intended user population) have no objections to reviews of un-anonymised notes for the purposes of the STROBE study

Participants expect the STROBE study to have a robust process in place for the reporting of clinical incidents, and that these should be communicated to the women concerned.
